# Effects of postoperative physical exercise rehabilitation on cardiorespiratory fitness, functional capacity and quality of life in patients with colorectal, breast, and prostate cancer – a systematic review and meta-analysis

**DOI:** 10.1007/s00432-024-06064-y

**Published:** 2024-12-24

**Authors:** Mailin Beyer, Christian Bischoff, Johannes Lässing, Ines Gockel, Roberto Falz

**Affiliations:** 1 Department of Rehabilitation and Sports Medicine, Hannover Medical School, Carl-Neuberg-Straße 1, 30627 Hannover, Germany; 2 Institute of Sports Medicine & Prevention, University Leipzig, Leipzig, Germany; 3 Department of Visceral, Transplant, Thoracic and Vascular Surgery, University Hospital Leipzig, Leipzig, Germany; 4 Institute of Sport Sciences, Martin-Luther-University Halle-Wittenberg, Halle, Germany

**Keywords:** Breast cancer, Colorectal cancer, Functional capacity maximum oxygen uptake, Morbidity, Physical exercise, Postoperative rehabilitation, Prostate cancer, Quality of life

## Abstract

**Purpose:**

The reduced cardiorespiratory fitness (CRF) and functional capacity following surgical procedures and during cancer treatments is a major risk factor for morbidity and mortality among patients with cancer. We aimed to assess the impact of endurance and combined resistance exercise interventions during the postoperative rehabilitation period for patients with colorectal, breast, and prostate cancer.

**Methods:**

A systematic search was conducted in MEDLINE Pubmed, Web of Science, and Cochrane Library until October 2023 for randomized controlled trials that assessed exercise interventions (aerobic/endurance; resistance or combined training) on postoperative patients with cancer. The trials evaluated the change in oxygen uptake (VO_2max_), six-minute walking distance (6MWD), quality of life (QoL), and fatigue.

**Results:**

Twelve studies, including 1298 patients, were part of this systematic review, and ten studies were included in the meta-analysis. Postoperative exercise interventions led to improvements in CRF and functional capacity (VO_2max_: MD 1.46 ml/kg/min; 95%-CI 0.33, 2.58; *p * = 0.01; 6MWD: MD 63.47 m; 95%-CI 28.18, 98.76; *p *= 0.0004, respectively) as well as QoL (0.91; 95%-CI 0.06, 1.76; *p *= 0.04). The quality of evidence was moderate to low.

**Conclusion:**

Postoperative exercise interventions could effectively improve CRF, functional capacity and QoL as shown in this meta-analysis. However, there is a lack of high-quality trials with a higher number of participants examining the effects of postoperative exercise in patients with colorectal, breast, and prostate cancer. There is an obvious need for long-term, cancer-specific exercise therapies and their evaluation in cancer care.

## Objectives

Each year, nearly 20 million people worldwide are newly diagnosed with cancer (Bray et al. 2024; Sung et al. 2021). In Germany, more than 4.5 million people currently live with or have survived a cancer diagnosis, with half of all cancers in Germany being breast, prostate, or colon carcinoma (Arndt et al. 2021). Early diagnosis and advancements in treatment have improved prognoses, creating a growing need to address unique health issues for cancer survivors (Campbell et al. 2019). Physical function plays an important role because cancer is strongly associated with aging. Those affected must deal not only with the effects of cancer treatment and its aftermath, such as the risk of developing heart disease that can accompany a cancer diagnosis but also with the aging effects of the organism (Curigliano et al. 2016; Arndt et al. 2021; Campbell et al. 2019; Scott et al. 2018; Miller et al. 2019). There are clear indications that regular physical exercise is an important additional component of cancer treatment to improve cancer-related health outcomes, particularly physical function or cardiorespiratory fitness (CRF), in secondary and tertiary prevention (Campbell et al. 2019). Friedenreich et al. (2017) proposed several potential beneficial biological mechanisms through which exercise, might delay tumor growth, lower the risk of metastatic disease, and enhance treatment efficacy. There are indications that exercise training or physical activity, which enhances CRF after a cancer diagnosis, is beneficial for overall survival and may help prevent a recurrence (Patel et al. 2019). This benefit varies based on the intensity levels of physical activity following the diagnosis. Overall, evidence from observational trials across diseases indicates that CRF and positive changes in CRF are inversely associated with the risk of all-cause mortality (Laukkanen et al. 2022; Kokkinos et al. 2023).

However, the most important measure of CRF, the oxygen uptake, is often calculated rather than directly measured, or just functional capacity measures are used. Published reviews and meta-analyses in this area have covered a variety of time periods in cancer treatment (e.g. prehabilitation or rehabilitation), outcomes, and exercise protocols (Cheng et al. 2017; Batalik et al. 2021; Baumann et al. 2012; Buffart et al. 2017; Courneya 2001; Cramer et al. 2014; Falz et al. 2022; Hilfiker et al. 2018; Kampshoff et al. 2014; McGettigan et al. 2020; Speck et al. 2010; Spence et al. 2010; Sweegers et al. 2018; Thomson et al. 2021). The wide range of findings makes it challenging to provide specific exercise recommendations (Cramer et al. 2014; Baumann et al. 2012; Lahart et al. 2018). Several reviews have concentrated on cancer-specific quality of life, surgical outcomes, as well as particular symptoms such as lymphedema, limited range of motion, or incontinence (McNeely et al. 2010; Hasenoehrl et al. 2020; Baumann et al. 2012; Thomson et al. 2021). A primary aim of an exercise intervention for cancer patients, however, is to effectively enhance CRF, as it is directly linked to improved morbidity and mortality outcomes. In summary, the basis for recommendations is limited, with the initial development of German guidelines for physical activity in patients with cancer currently underway (German Cancer Society, 2019; German Cancer Society, 2021; German Cancer Society, 2024).

Currently, there is no meta-analysis available that examines the impact of aerobic and combined aerobic and resistance exercise interventions on objectively measured CRF, as indicated by oxygen uptake, during the postoperative period in patients with colorectal, breast, and prostate cancer. Therefore, this systematic review and meta-analysis aim to evaluate the effect of postoperative exercise interventions on CRF by measuring oxygen uptake and functional capacity evaluated by the six-minute walk test (6MWD). Additionally, we focused on the three most common cancer entities, which show significant evidence of exercise effects on mortality and morbidity (Patel et al. 2019; Arndt et al. 2021). We also assessed quality of life and changes in fatigue.

## Methods

### Search strategy

This review was conducted and recorded in accordance with the Cochrane systematic review guidelines and Preferred Reporting Items for Systematic Reviews and Meta-Analysis Checklist (PRISMA) (Page et al. 2021). It was also prospective registered with the International Prospective Register of Systematic Reviews (PROSPERO 2022; CRD42022355287). Two of the authors (CB, MB) performed a systematic literature search within the electronic databases PubMed (NCBI; all fields), Cochrane Library (Wiley; all fields), and Web of Sciences (https://www.webofscience.com/;all fields) initially on 1 April 2022 and rerun on 1 October 2023. The search included terms such as ‘breast cancer postoperative exercise rehabilitation’ OR ‘prostate cancer postoperative exercise rehabilitation’ OR ‘colorectal cancer postoperative exercise rehabilitation’ OR ‘colon cancer postoperative exercise rehabilitation’ OR ‘rectal cancer postoperative exercise rehabilitation’, while excluding ‘review’ and ‘meta-analysis’, without any limits. Additionally, we screened also studies through the reference lists in relevant articles and reviews. We did not search for grey literature or seek additional studies by contracting authors.

### Study selection

**Fig. 1:**
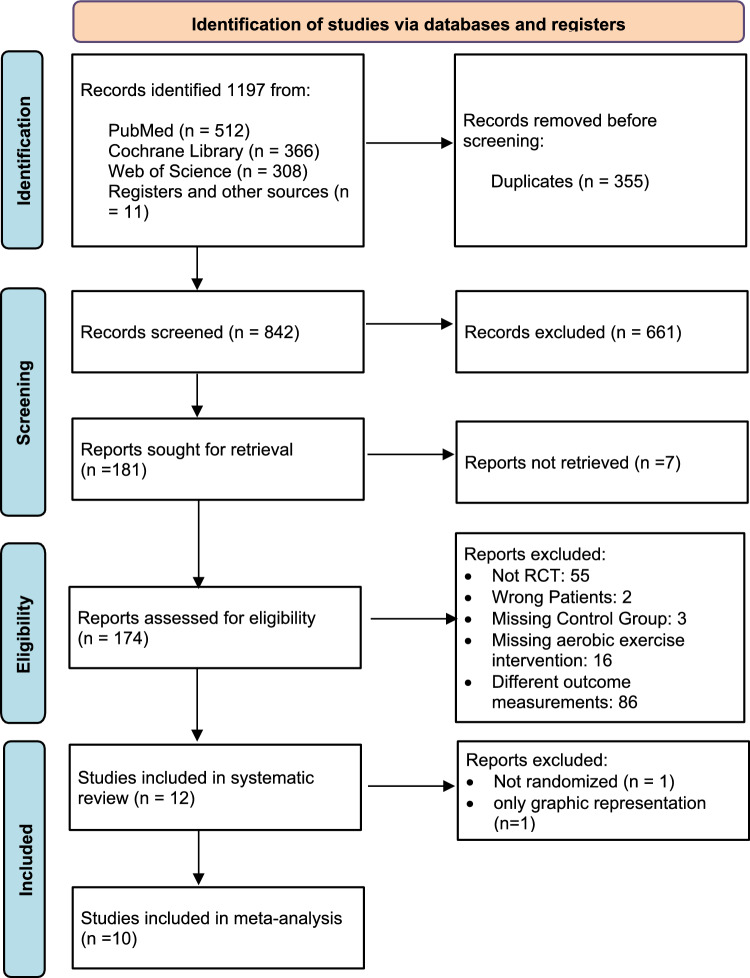
PRISMA flow chart of included and excluded studies in this systematic review and meta-analysis (Page et al. 2021)

Three independent reviewers (CB, MB, JL) screened potentially eligible articles after removing duplicates and reviewing for our set inclusion criteria. Disagreements were resolved by consensus. This review included RCTs and prospective controlled trials that examined the functional outcome effects (VO_2max_, 6MWT, 12MWT) of an exercise intervention on adults with resected colorectal, breast, or prostate cancer. Detailed inclusion criteria are found in Table 1. Our systematic literature search process is depicted in Fig. 1.

**Table 1:** Inclusion criteria according to PICOS schema for systematic review and meta-analysis

Category	Description
Population	adults diagnosed with colorectal, breast, or prostate cancer resection less than 24 months before intervention start
Intervention	aerobic/endurance or combined cardiovascular and resistance exercise ≥ 2 weeks
Comparison	at least one comparison group receiving usual/regular care
Outcome	Primary: measured VO_2max_ or 6MWD or 12MWD Secondary: quality of life or fatigue
Publication language	English
Study design	RCTs for meta-analysis, as well as Quasi-randomized trials for systematic review

### Data extraction

Study inclusion was initially decided by CB and MB and discussed with the senior author RF. The selected studies were organized into narrative analysis in Tables 2 and 3 based on the functional outcome measured. These tables contain details from the selected studies, including sample size, cancer entity, type of postoperative exercise intervention, details of the exercise intervention (such as training frequency, session time, and intensity), age of participants, duration of intervention, adherence to the intervention, and main results.

### Quality assessment (risk of bias and quality of evidence)

The methodological quality of each study was assessed independently by two authors (CB and MB) using the Cochrane risk of bias tool ROB2 (Higgins et al. 2011). Five components of bias were evaluated: bias arising from the randomization process; bias due to deviations from intended interventions: bias due to missing outcome data; bias in the measurement of the outcome; and bias in the selection of the reported result. The tools evaluate criteria such as randomization method; allocation concealment; baseline comparability of study groups; blinding and completeness of follow-up. Trials were categorized as having low (green circle), high (red circle), or unclear (yellow circle) risk of bias. Publication bias was assessed visually and with a funnel graph.

The Grading of Recommendations, Assessment, Development, and Evaluation (GRADE) approach was used to interpret and evaluate the quality of evidence (Guyatt et al. 2011a). The overall quality of evidence for each pooled estimate was initially considered “high”, and could be downgraded by 1 level for each of the following 5 criteria: risk of bias (any of the trials included in the analysis showed “high” or “unsure”) (Guyatt et al. 2011f), inconsistency (large heterogeneity among trials, I^2^ > 50%) (Guyatt et al. 2011d), imprecision of evidence (< 400 participants for each comparison) (Guyatt et al. 2011c), indirectness of effect estimates (indirectness of population, outcomes or intervention) (Guyatt et al. 2011b), and potential reporting bias (which was assessed by an asymmetry in the funnel plot) (Guyatt et al. 2011e).

### Data synthesis and statistical analysis

The quantitative synthesis was performed using RevMan 5 (Review Manager 5 software, Version 5.4, The Cochrane Collaboration, 2020). In cases of missing data, study authors were contacted. Continuous outcomes were analyzed using the random-effects model to calculate the weighted mean difference and 95% confidence interval, which were visualized in forest plots. We selected the random model due to the expected heterogeneity from the varying exercise interventions. The functional outcome effects were determined by extracting data directly from the included study or calculating them from the mean and 95% confidence intervals. In cases where the mean and standard deviation of the change from baseline were not reported in the papers, we used specific equations to calculate them or reached out to the authors for the original database. The correlation coefficient was calculated as described by Higgins et al. (2024).

Mean_chance_ = Mean_endpoint_ - Mean_baseline_.

SD_change_ = $$\:\sqrt{\left(\varvec{S}\varvec{D}baseline\right)2+\left(\varvec{S}\varvec{D}endpoint\right)2+2xrx\varvec{S}\varvec{D}baselinex\varvec{S}\varvec{D}\text{endpoint}}$$

The quality of life (QoL) and fatigue variables were calculated using standardized mean difference with standard error, based on the diversity of questionnaire surveys used. QoL data were extracted from the Functional Assessment of Cancer Therapy General Scale (FACT-G), the Functional Assessment of Cancer Therapy-Anemia Scale (FACT-AN), or the subscale of the European Organization for Research and Treatment of Cancer Quality of Life Questionnaire C30 (EORTC QLQ-C30). The fatigue variable was derived from the fatigue subscales of the FACT survey or from the EORTC QLQ-C30’s fatigue subscale.

I^2^ was used to assess statistical heterogeneity. We categorized the results as follows: less than 25% - low heterogeneity; to 25% and 75% - potentially moderate heterogeneity; over 75% - considerable heterogeneity. Random effects models were employed to calculate overall effects, and forest plots to depict estimates.

For all statistical analyses, *p *< 0.05 was considered statistically significant.

## Results

In October 2023, the search identified 1187 papers. Among these, 355 were duplicates and removed before the initial screening. Eleven additional papers were obtained from other sources, such as citations in screened publications. This brought the total number of articles and reports screened to 842. At first glance, 12 papers met our inclusion criteria (Anderson et al. 2012; Bøhn et al. 2021; Cantarero-Villanueva et al. 2016; Courneya et al. 2003, 2007; Falz et al. 2023; Lee et al. 2017; Lin et al. 2013; Murtezani et al. 2014; Mutrie et al. 2007; Travier et al. 2015; Van Vulpen et al. 2016). One of these 12 studies only described their functional outcome graphically. After consulting with the authors about the original dataset, it was decided to exclude this study (Bøhn et al. 2021). Another study was excluded due to the lack of a randomization process (Lin et al. 2013). In total, the meta-analysis included ten studies with a combined total of 1198 randomly assigned study participants (including dropouts). Half of the included studies measured maximum oxygen uptake (VO_2max_) directly during incremental exercise tests. Three trials used the 6-minute walking distance (6MWD) as a measure, and two trials used the 12-minute walking distance (12MWD) as functional outcome for cardiovascular assessment. The characteristics and main outcome of the studies included in our systematic review are summarized in Table 2. Studies excluded from the meta-analysis are indicated.

**Table 2:** Summary of study characteristics and outcomes regarding postoperative rehabilitation programs involving exercise interventions

Study	Cancer	Additional treatments/influential factors	Study design	Sample size (IG/CG)	Age, years IG vs. CG	Exercise intervention/ control	Drop-outs IG vs. CG	Objectvely measured outcomes IG vs. CG
Anderson et al. 2012	Breast	Lymph-edema education	RCT	104 (52/52)	m (R) 53.6 (32–82)	Specific lymphedema intervention + 60 min combined moderate aerobic and resistance training two times per week vs. usual care + information about preventing lymphedema, nutrition and physical activity	9 vs.13	*∆6MWD [m]*^*B*^: 54.0 ± 183.43 vs. 20.9 ± 165.78 (*p* = 0.0098)
Bøhn et al. 2021^*^	Ductal carcinoma in situ grade 3, Breast stage I, II		RCT	55 (29/26)	55.7 ± 7.8 54.3 ± 7.7	Group training sessions (moderate to high intensity aerobic endurance training) for 60 min two times per week + 120 min exercise at home vs. standard care + voluntary exercise	6 vs. 3	Significant change in mean VO_2max_ [ml/kg/min] between the groups. Data only reported graphically
Cantarero-Villanueva et al. 2016	Colon		RCT	46 (23/23)	57.5 ± 8.0 62.3 ± 7.9	Three sessions 90 min Core stabilization exercises weekly	9 vs. 8	*∆6MWD [m]*: 79.7 ± 106.33^A^ vs. 4.9 ± 106.02^A^
Courneya et al. 2003	Breast	Post-menopausal patients	RCT	50 (24/26)	59 ± 5 58 ± 6	Supervised aerobic exercise vs. no training during intervention period	1 vs. 2	*∆VO*_*2max*_*[ml/kg/min]*: + 2.7 ± 2.6 vs. −0.6 ± 1.7 (*p* < 0.001) *∆PPO [w]*: + 14.2 ± 18.7 vs. −16.5 ± 18.6 (*p* < 0.001)
Courneya et al. 2007	Breast	Undergoing chemotherapy 2 intervention groups	RCT	242 (78/82/ 82) (AET/ RET/CG)	m ^®^ AET: 49.5 (25–76) RET: 49 (30–75) CG: 49 (26–78)	Aerobic exercise AET vs. resistance exercise RET vs. no newly initiated exercise program CG	AET: 7 RET: 5 CG: 9	*∆VO*_*2max*_*[ml/kg/min]*^*B*^: AET 0.2 ± 14.16 vs. RET − 1.4 ± 11.83 vs. CG −1.6 ± 11.25 (AET vs. CG: *p* = 0.004; AET vs. RET: *p* = 0.031)
Falz et al. 2023	Colorectal, Breast, Prostate		RCT	122 (62/60)	54.4 ± 11 54.6. ± 12	Home-based body-weight strength-endurance training vs. general information about lifestyle changes and physical activity and wearables for activity tracking	14 vs. 12	*∆VO*_*2max*_*[ml/min/kg]*: 1.82 ± 2.71 vs. 0.66 ± 3.5 *∆PPO [w]*: 10.2 ± 16.2 vs. 6.3 ± 18.7
Lee et al. 2017	Colorectal		RCT	123 (62/61)	56.3 ± 9.7 56.3 ± 9.9	Daily home-based endurance and resistance activity vs. maintaining usual daily activity	11 vs. 13	*∆6MWD [m]*^*B*^: 25.2 ± 149.60 vs. −9.2 ± 142.91
Lin et al. 2013^*^	Colorectal, stage II - III	Undergoing chemo-therapy	Controlled trial	45 (21/24)	59.0 ± 9.5 54.3 ± 10.6	Moderate aerobic/resistance exercise for 60 min two times per week vs. usual care	1 vs. 5	*∆6MWD [m] mean (95% CI)*: 58.93 (40.59–77.27) vs. 44.60 (19.73–69.48) (*p* = 0.353) *∆Physical activity [MET] mean (95% CI)*: 1996.74 (328.96–3664.52) vs. −266.96 (−1030.57–496.66) (*p* = 0.01)
Murtezani et al. 2014	Breast		RCT	73 (37/36)	53 ± 11 51 ± 11	Supervised moderate intensity aerobic exercise vs. sedentary lifestyle	7 vs. 4	*∆12MWD[m]*^*B*^: 75.5 ± 164.35 vs. 9.1 ± 168.51
Mutrie et al. 2007	Breast		RCT	201 (99/102)	51.3 ± 10.3 51.8 ± 8.7	45 min combined exercise three times per week vs. usual care + information about exercise after cancer diagnosis	19 vs. 7	*∆12MWD[m]*^*B*^: 138 ± 343.62 vs. 9 ± 442.12
Travier et al. 2015	Breast	During adjuvant treatment	RCT	204 (102/102)	49.7 ± 8.2 49.5 ± 7.9	Supervised aerobic and strength exercise intervention	15 vs. 25	*∆VO*_*2max*_*[ml/kg/min]*: −2.8 ± 4.38^A^ vs. −3.2 ± 5.15^A^
Van Vulpen et al. 2016	Colon	Undergoing Chemotherapy	RCT	33 (17/16)	58.1 ± 10.3 58.1 ± 9.6	Supervised aerobic and resistance exercise vs. usual care	2 vs. 3	*Male Participants*: *∆VO*_*2max*_*[ml/kg/min]*: −0.7 ± 3.7^A^ vs. 0.5 ± 3.5^A^ *Female Participants*: *∆VO*_*2max*_*[ml/kg/min]*: 0.7 ± 3.35^A^ vs. −1.0 ± 3.22^A^

Mean m and standard deviation SD are presented. Other data (median MD, 95% Confidence interval 95%CI) are marked. Order of groups in the columns: Sample size; Age and Main Outcomes: IG vs.CG

*IG* intervention group, *CG* control group, *RCT* randomized controlled trial, *6MWD* six minute walk distance, *VO2* oxygen uptake, *PPO* peak power output, *MET* metabolic equivalent of task, *EORTC QLQ-C30* European Organization for Research and Treatment of Cancer Core Quality of Life Questionnaire

^*^Excluded from meta-analysis

^A^ SD calculated by 95% CI

^B^ mean calculated by post-pre + SD calculated by (√(SD_base_^2^+SD_final_^2^+2*0,88*SD_base_*SD_final_))

### Study characteristics

Seven of the twelve remaining studies focused on patients with breast cancer only (Anderson et al. 2012; Bøhn et al. 2021; Courneya et al. 2003, 2007; Murtezani et al. 2014; Mutrie et al. 2007; Travier et al. 2015). Two studies assessed patients with colon cancer (Cantarero-Villanueva et al. 2016; van Vulpen et al. 2016), and two studies examined patients with colorectal cancer (Lee et al. 2017; Lin et al. 2013). Only one study assessed the effectiveness of a postoperative training intervention in mixed patients with colorectal, breast and prostate cancer (Falz et al. 2023). In our meta-analysis, patients with colon and colorectal cancer are evaluated together and summarized as ‘colorectal´. The analysis examined a total of 1013 postoperative patients with breast cancer, including dropouts, with 546 undergoing some sort of exercise intervention. In the collective group of colorectal patients, there were 266 individuals, including dropouts, of whom 133 participated in the intervention groups. 45 patients with prostate cancer were examined by Falz et al. (2023), 23 of whom underwent an exercise intervention.

The primary outcomes focused on improving CRF or functional capacity, which was measured in all studies using oxygen uptake and the 6MWD/12MWD. Some studies also reported other outcomes such as arm volume or lymphedema (Anderson et al. 2012; Courneya et al. 2007), blood parameters related to hemostasis, inflammation or hormones (Bøhn et al. 2021; Falz et al. 2023; Lee et al. 2017), different strength and mobility parameters (Cantarero-Villanueva et al. 2016; Lin et al. 2013; Mutrie et al. 2007), anthropometric or body composition data (Cantarero-Villanueva et al. 2016; Courneya et al. 2007; Falz et al. 2023; Murtezani et al. 2014; Mutrie et al. 2007; Van Vulpen et al. 2016), various questionnaires or subscales (Courneya et al. 2007; Lin et al. 2013; Mutrie et al. 2007; Travier et al. 2015; Van Vulpen et al. 2016), chemotherapy completion rate (Courneya et al. 2007; Van Vulpen et al. 2016) cardiovascular parameters (Falz et al. 2023), and overall physical activity (Falz et al. 2023; Lin et al. 2013; Mutrie et al. 2007). Subjects in four studies (Courneya et al. 2007; Lin et al. 2013; Travier et al. 2015; Van Vulpen et al. 2016) were undergoing adjuvant chemo- and/or radiotherapy treatment during the intervention. Surgical procedures are reported in nine of the 12 studies (Anderson et al. 2012; Bøhn et al. 2021; Cantarero-Villanueva et al. 2016; Courneya et al. 2003, 2007; Murtezani et al. 2014; Mutrie et al. 2007; Travier et al. 2015; Van Vulpen et al. 2016), but the categories vary across the trials, and subgroup evaluations are not included.

Cantarero-Villanueva et al. (2016) and Courneya et al. (2003) reported adverse events by groups, and the absolute numbers of adverse events in the intervention groups were twice as high in Cantarero-Villanueva et al. (2016) (IG: 2 vs. CG: 1) and 20.8% vs. 7.1% IG vs. CG in Courneya et al. (2003). Moreover, two trials reported on overall numbers of adverse events (Courneya et al. 2007; Falz et al. 2023). Reasons for adverse events included postoperative ventral hernias (Cantarero-Villanueva et al. 2016), lymphedema, other medical complications not related to the intervention, or accidents (Courneya et al. 2003; Falz et al. 2023) and minor medical problems from exercise testing (Courneya et al. 2007). Overall dropouts were very similar across groups (106 IG vs. 104 CG).

### Exercise interventions

The exercise interventions varied in terms of types and prescribed intensity. Six trials involved combined aerobic and resistance exercises (Anderson et al. 2012; Cantarero-Villanueva et al. 2016; Courneya et al. 2007; Lee et al. 2017; Mutrie et al. 2007; van Vulpen et al. 2016). Four trials focused solely on aerobic exercise (Bøhn et al. 2021; Courneya et al. 2003; Murtezani et al. 2014; Travier et al. 2015). Falz et al. (2023) used exclusively body-weight strength-endurance training. The duration of the different interventions varied ranging from two months (Cantarero-Villanueva et al. 2016) to one year (Anderson et al. 2012). Home-based training sessions ranged from two to three times per week (Falz et al. 2023) and daily exercising (Lee et al. 2017). Six trials had supervised training sessions two to three times per week (Cantarero-Villanueva et al. 2016; Courneya et al. 2003, 2007; Murtezani et al. 2014; Travier et al. 2015; van Vulpen et al. 2016). Anderson et al. (2012) and Mutrie et al. (2007) combined supervised and home-based sessions three times per week.

The intensity of aerobic exercise was monitored through heart rate ranges that are individually defined by maximum heart rate or the VO_2max_ (Bøhn et al. 2021; Courneya et al. 2003, 2007; Lee et al. 2017; Lin et al. 2013; Murtezani et al. 2014; Mutrie et al. 2007; Travier et al. 2015; Van Vulpen et al. 2016). The intensity specifications for resistance training reveal different percentage ranges of a single repetition maximum (Anderson et al. 2012; Courneya et al. 2007; Travier et al. 2015; Van Vulpen et al. 2016). Other scales, such as the rating of perceived exertion (RPE), Borg Category-Ratio scale (CR10), and targeted metabolic equivalent of task (MET) values, were also used (Anderson et al. 2012; Cantarero-Villanueva et al. 2016; Falz et al. 2023; Lee et al. 2017).

The results of eight of the twelve trials included information about adherence, with varying methods of measurement. Some trials measured participation rate in the exercise classes offered (Anderson et al. 2012; Cantarero-Villanueva et al. 2016; Courneya et al. 2003, 2007; Travier et al. 2015; Van Vulpen et al. 2016), while others measured the percentage of patient’s rate fulfilling100% of the recommendations (Falz et al. 2023; Lee et al. 2017). Due to these diverse measurement methods, it wasn´t possible to accurately assess differences. However, it was noted that 56.4% (Falz et al. 2023) and 86.2% (Lee et al. 2017) of patients completed all recommended exercises. Additionally, the intervention participation rate varied from 71.2% (Anderson et al. 2012) to 98.4% (Courneya et al. 2003).

**Table 3:** Characteristics of exercise program in included RCT´s

Study	Description of exercise intervention	Intervention start post-surgery	Duration of rehabilitation	Training frequency [per week]	Session duration	Overall training sessions	Intensity / control of intensity	Adherence in training sessions / (serious) Adverse events
Anderson et al. 2012	a) supervised strength and cardiovascular training b) supervised and home-based strength and cardiovascular training c) voluntarily supervised training + home-based training	a) 4–12 weeks b) after a c) after b	a) 12 weeks b) 12 weeks c) 24 weeks	a) 2x b) > 1x c) voluntarily	65 min		a) Resistance training: 50% established 1RM weekly increased by 1–5lbs; cardiovascular training: 14–16 RPE	71.2% R:0–97
Bøhn et al. 2021*	a) Group-based moderate to high aerobic and strength exercise b) home-based exercise	21–28 days	48 weeks	a) 2x b) 120 min	a) 60 min		VO_2max_ not described in detail	
Cantarero-Villanueva et al. 2016	Core stabilization exercises	12.0 ± 7.4 months 14.6 ± 10.0 months	8 weeks	3x	20–30 min	22.0 ± 1.1	RPE	88.36% / IG: 2, CG: 1
Courneya et al. 2003	Supervised cycle ergometry sessions	IG: 14 ± 6 months CG: 14 ± 7 months	15 weeks	3x	Wk1-3: 25 min; increased by 5 min every third week	44.3	5 min warm-up + cool-down 50% of VO_2max_; training intensity at 70–75% of VO_2max_	98.4% / Adverse events: IG: 20,8% CG: 7.1%
Courneya et al. 2007	a) supervised aerobic exercise (AET) b) supervised resistance exercise (RET) − 9 exercises á 2 sets á 8–12 reps	Not given	Mean: 17 weeks	3x	a) Wk1-3: 25 min; increased by 5 min every third week		a) 60% of VO_2max_ wk1-6, 70% of VO_2max_ wk 7–12, 89% of VO_2max_ wk > 12 b) 60–70% of 1RM increased by 10% if 12 reps completed	AET: 72% RET: 68.2% / Adverse events: 2
Falz et al. 2023	Home-based, bodyweight strength-endurance training	Not given	24 weeks	2-3x	30 min	1.5 sessions per week (36 overall sessions)	Entity-specific, individually performance adapted and heart rate limited; targeted 5–8 CR10 scale	56.4% Adverse events: 18
Lee et al. 2017	a) home based endurance exercise b) home based resistance exercise	IG: 10.7 ± 8.8 CG: 8.8 ± 7.2	12 weeks	Daily	b) 30 min		3000 steps with HR > 65% of estimated HR_max_, 18–27 MET-hours per week	> 18 MET-hours: 86.3% 27 MET-hours: 74.5%
Lin et al. 2013*	Group based supervised combined aerobic and resistance exercise	37.8 (16.4) days	12 weeks	2x	40–60 min	24	Increasingly from 40–75 of HR_max_	73% (17.3)
Murtezani et al. 2014	supervised group moderate intensity aerobic exercise program	19.0 ± 6.9 vs. 19.1 ± 4.8	10 weeks	3x	25 min		Increasingly from 50–75 of estimated HR_max_ and from 25 min to 45 min	
Mutrie et al. 2007	a) group based supervised diverse exercise b) home based exercise session	Not given	12 weeks	a) 2x b) 1x	45 min		50–72% of age adjusted HR_max_	
Travier et al. 2015	supervised combined aerobic and muscle strength training	within 10 weeks post diagnosis	18 weeks	2x	60 min		different aerobic methods according to HR on ventilatory threshold + strength exercises between 45–75% of 1RM (increasing repetitions or intensity)	83% (IQR: 69–91%)
Van Vulpen et al.^2016^	supervised combined aerobic and muscle strength training	within 10 weeks post diagnosis	18 weeks	2x	60 min		different aerobic methods according to HR on ventilatory threshold + strength exercises between 45–75% of 1RM (increasing repetitions or intensity)	89% (IQR: 72–97%)

Mean and standard deviation are presented. Other data (median MD, 95% Confidence interval 95%CI; interquartile range IQR; Range R) are marked

*HR max* maximal reached heart rate, *1RM* one repetition maximum, *RPE* rating of perceived exertion, *CR-10* Category-Ratio Scale anchored at 10, *MET* metabolic equivalent of task, *MAP* maximal aerobic power

*Excluded for meta-analysis

## Control groups

A total of 610 patients were assigned to control groups (CG). These patients received standard care in three studies (Lee et al. 2017; Travier et al. 2015; van Vulpen et al. 2016). In addition, they were provided with extra information about physical activity during cancer rehabilitation in three other trials (Anderson et al. 2012; Cantarero-Villanueva et al. 2016; Mutrie et al. 2007). Both groups used technical devices to monitor daily activities, and were given access to an informational platform about physical activity during postoperative cancer treatment in Falz et al. (2023). The remaining three trials instructed their CG participants not to initiate new exercises during the intervention period (Courneya et al. 2003, 2007; Murtezani et al. 2014).

### Risk of Bias and quality of evidence for each outcome measure considered following GRADE assessment

**Fig. 2:**
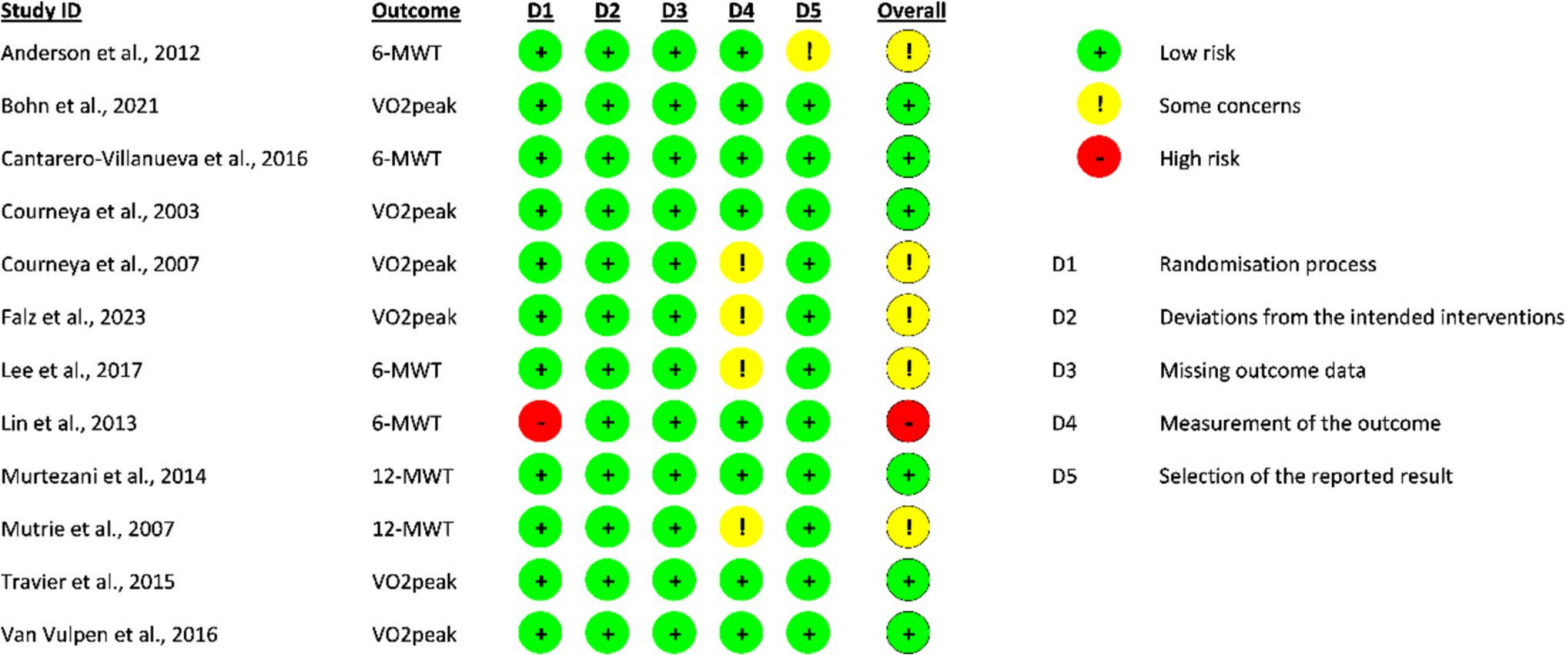
Cochrane risk of bias assessment of randomized controlled trials included in meta-analysis

In our assessment of bias, six studies (Bøhn et al. 2021; Cantarero-Villanueva et al. 2016; Courneya et al. 2003; Murtezani et al. 2014; Travier et al. 2015; van Vulpen et al. 2016) were found to have a low risk of bias. Five studies raised concerns about bias related to how outcomes were measured (Courneya et al. 2007; Falz et al. 2023; Lee et al. 2017; Mutrie et al. 2007) or the reported results (Anderson et al. 2012) (Fig. 2). Only one study was excluded from meta-analysis due to a high risk of bias in the randomization process (Lin et al. 2013), as was the study by Bøhn et al. (2021) due to missing result data.

The GRADE assessment for the quality of evidence showed low quality of evidence for VO2max and QoL (downgraded due to risk of bias, inconsistency) and moderate quality of evidence for 6-MWD and Fatigue (downgraded due to risk of bias) Table 4. As none of the comparisons included 10 or more studies, publication bias could only assessed in Funnel plots visually.

**Table 4:** GRADE assessment for the certainty of evidence

	No trials	Risk of bias	Inconsistency	Indirectness	Imprecision	Publication bias	No of patients (IG/CG)	Effect SMD (95% CI)	quality
VO_2max_	6	serious	Serious	Not serious	Not serious	Undetected	283/288	0.42 higher (0.04–0.79 higher)	Low
6-MWD	5	serious	Not serious	Not serious	Not serious	Undetected	273/274	0.44 higher (0.27–0.61 higher)	Moderate
QoL	6	serious	Serious	Not serious	Not serious	Undetected	320/324	0.91 higher (0.06–1.76 higher)	Low
Fatigue	4	serious	Not serious	Not serious	Not serious	Undetected	221/228	0.22 higher (− 0.07 lower–0.59 higher)	moderate

VO2max maximal oxygen uptake; 6 or 12-MWD 6- or 12-minute walk distance

*QoL* quality of life, *IG* intervention group, *CG* control group, *SMD* standard mean difference, *CI* confidence interval

### Meta-analysis of main outcome parameters

**Fig. 3:**
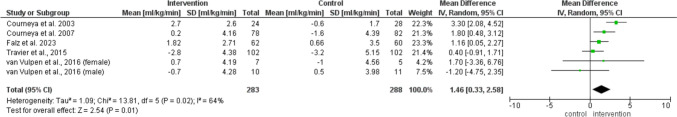
Meta-analysis of the change in VO_2max_ after postoperative exercise

Five studies measured VO_2max_ (Courneya et al. 2003, 2007; Falz et al. 2023; Travier et al. 2015; van Vulpen et al. 2016. The analysis of mean change in VO_2max_ showed a significantly higher improvement (MD 1.46 ml/kg/min; 95% CI 0.33, 2.58; *p* = 0.01; *I*_*2*_ = 64%) in the IG (Fig. 3).

**Fig. 4:**
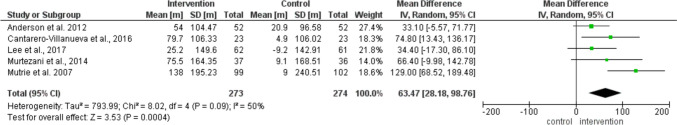
Meta-analysis of the change in walking distance in 6MWT or 12MWT after postoperative exercise

The evaluation of the five studies examining 6MWD as a functional capacity marker (Anderson et al. 2012; Courneya et al. 2003; Cantarero-Villanueva et al. 2016; Lee et al. 2017; Murtezani et al. 2014; Mutrie et al. 2007) confirms the positive effect. The IG increased their walking distance significantly more than the CG (MD 63.47 m; 95% 28.18, 98.76; *p* = 0.0004; *I*_*2*_ = 50%) (Fig. 4).

**Fig. 5:**
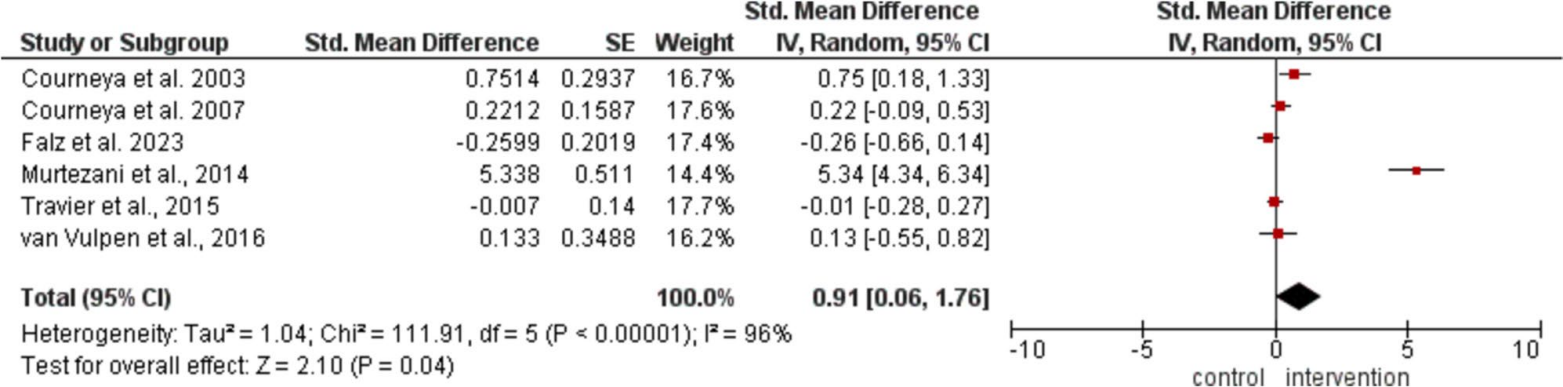
Meta-analysis of the change in quality of life after postoperative exercise

Quality of life was assessed in six of the included trials (Falz et al. 2023; Courneya et al. 2003, 2007; Murtezani et al. 2014; Travier et al. 2015; van Vulpen et al. 2016), where the IG demonstrated a significantly higher increase compared to the CG (MD 0.91; 95% CI 0.06, 1.76; *p* = 0.04; *I*_*2*_ = 96%) (Fig. 5).

**Fig. 6:**
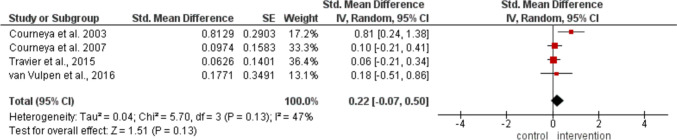
Meta-analysis of the change in fatigue after postoperative exercise

Regarding fatigue during the intervention period, no significant differences were observed between groups in the four trials (Courneya et al. 2003, 2007; Travier et al. 2015; van Vulpen et al. 2016) (MD 0.22; 95% CI −0.07, 0.50; *p* = 0.13; *I*_*2*_ = 47%) as shown in Fig. 6.

## Discussion

The current systematic review included 12 studies that investigate the impact of exercise interventions on patients with colorectal, breast, and prostate cancer within two years after cancer surgery. The reported findings yield evidence of the positive impact of exercise interventions on CRF in patients during postoperative rehabilitation. These results underline the latest research, which suggests that encouraging patients with cancer to engage in active exercise programs can improve their CRF and overall well-being. Considering the strong evidence about the inverse effect of CRF on the relative risk of all-cause and cancer-cause mortality, even small improvements are beneficial, particularly for patients with colorectal, breast, and prostate cancer (Jensen et al. 2017; Laukkanen et al. 2022; Kokkinos et al. 2023; Patel et al. 2019; Schmid and Leitzmann 2015).

The 6MWD is a common method to assess functional capacity in patients with heart and lung diseases. Studies have shown that clinically significant improvements in these patients typically range from 14 to 42 m (Moutchia et al. 2023; Bohannon and Crouch 2017; Granger et al. 2015). However, it´s worth noting that while these trials demonstrate an important increase, they do not provide information on patients with colorectal, breast, and prostate cancer. Further research is necessary to validate these findings in those specific patient populations.

The small number of trials in our review prevented us from conducting a subgroup analysis of results. Research interest in exercise intervention trials for patients with cancer and survivors has increased over the last three decades, resulting in the identification of 842 trials in the systematic literature search. However, we had to exclude most of these trials for various reasons, including the lack of randomization or a high risk of bias. However, is that other meta-analyses evaluating cancer rehabilitation interventions did not exclude such trials (Høeg et al. 2019; Bradt et al. 2011). Additionally, while predicted CRF is frequently reported, the trials had to be excluded (Bourke et al. 2011; Daley et al. 2007; De Luca et al. 2016; Nusca et al. 2021; Pinto et al. 2005) whereas directly measured VO_2max_ is rarely assessed.

CRF is not closely associated with the acute symptoms in postoperative patients with colorectal, breast, and prostate cancer, such as incontinence or reduced range of shoulder motion. Numerous trials focus on short-term side effects, leading to the exclusion of 86 trials (e.g., Wennerberg et al. 2023; Schrempf et al. 2023; Min et al. 2023; Shu et al. 2023; Park et al. 2023) However, the long-term benefits of improved CRF should not be neglected, indicating the need for further research. Some trials were excluded due to missing information about the medical history of the subjects, lack of randomization, and incompletely reported outcome measures (Alibhai et al. 2019; Battaglini et al. 2007; Leclerc et al. 2017; Schwartz and Winters-Stone 2009; Segal et al. 2009). These methodological differences may explain variations between some trials.

Courneya et al. (2003) found that the most significant improvements in VO_2max_ in patients with breast cancer were achieved through individually tailored aerobic exercise using cycle ergometry after completing chemotherapy or radiation therapy. This contrasted with other trials where a smaller effect was observed, possibly due to the exclusive use of an aerobic cycle ergometry exercise instead of combined aerobic and resistance exercise, which was sometimes performed during chemotherapy. The results of the only colon cancer trial included in this evaluation (Van Vulpen et al. 2016) show differences between female and male patients. Surprisingly, male patients experienced decreased VO_2max_ during the intervention period, a phenomenon the authors could not explain.

Only one of the six trials assessing quality of life reported a conspicuous effect (Murtezani et al. 2014). The methods used in that trial, such as patient characteristics or assessments, are comparable to those of the other trials, and that difference is not explained.

We found that the overall effects on quality of life and fatigue were lower than anticipated. Most published reviews of comprehensive programs for patients with cancer and survivors reported significant positive effects on quality of life or fatigue (Baumann et al. 2012; Buffart et al. 2017; Cheng et al. 2017; Hilfiker et al. 2018; Speck et al. 2010; Sweegers et al. 2018). However, some reviews yielded ambiguous results on fatigue and quality of life (Batalik et al. 2021; Spence et al. 2010) and even indicated no significant effect of exercise interventions on patients with colorectal cancer (Cramer et al. 2014). The methods in the mentioned reviews vary, and there is still significant research interest in the effects of exclusive physical exercise interventions on fatigue and quality of life. Further high-quality prospective randomized trials with adequate participant numbers are urgently needed to address these two relevant outcome parameters.

We were surprised that the time, duration, intensity, and frequency variations across the trials did not seem to noticeably impact results. The shortest interventions (Cantarero-Villanueva et al. 2016: 8 weeks; Murtezani et al. 2014: 10 weeks) showed a similar increase in 6MWD compared to the longer interventions (Anderson et al. 2012: 48 weeks), despite no disparities in the intensity and frequency of the exercises. However, a meta-analysis must confirm this observation, including more trials than ours. The level of adherence we noted in the trials included in our review did not differ from that observed in other meta-analyses (Batalik et al. 2021; Falz et al. 2022). The number of trials reporting (serious) adverse events is too limited to conduct robust evaluations or draw definitive conclusions. The author describes the highest absolute number of reported serious adverse events as unrelated to the intervention (Falz et al. 2023).

## Limitations

One major limitation of this meta-analysis is the absence of RCTs investigating exercise training during postoperative periods in patients with cancer. This limitation is consistent with those mentioned in other reviews (Batalik et al. 2021; Cheng et al. 2017; Cramer et al. 2014; Spence et al. 2010). Second, the methodological quality of trials has not shown improvement, and there has been persistent insufficient reporting of exercise interventions, according to Spence et al. (2010). Other notable issues include inconsistent reporting of Inclusion criteria, missing or inadequately described patient characteristics, and insufficient information on chemotherapy or radiotherapy during the intervention period, outcome measurement methods, and results. Third, comparing trial results becomes much more difficult when interventions and subjects are very specific. For example, when only post-menopausal or anemic patients with breast cancer or combined pharmacological and exercise interventions are considered (Courneya et al. 2008; Dieli-Conwright et al. 2018). Fourth, it was impossible to conduct subgroup analyses targeting exercise duration, training intensity, or type of exercise (such as aerobic vs. resistance training; supervised vs. non-supervised) due to the lack of differentiated patient groups or insufficient available data.

## Conclusions

Based on the available evidence from RCTs, this meta-analysis demonstrated post-operative exercise interventions in patients with cancer cardiorespiratory fitness, functional capacity, and quality of life. The period after surgery seems to be a feasible time for exercise interventions to support recovery and enhance patient outcomes. The potential to enhance patients’ cardiorespiratory fitness and functional capacity may lower morbidity and overall mortality. However, there is a need for high-quality postoperative exercise trials to analyze different types of interventions, such as home-based or supervised exercise and aerobic or resistance training. Evidence from these studies could help develop specific exercise guidelines for patients with cancer during and after surgical, pharmacological, and/or radiation therapy, as modern tumor therapy often involves multimodal treatments.

## Data Availability

No datasets were generated or analysed during the current study.
